# Employment satisfaction among public health and preventive medicine graduates and associated factors: a cross-sectional survey at Guangxi Medical University

**DOI:** 10.3389/fpubh.2026.1985186

**Published:** 2026-10-01

**Authors:** Qiyini Ma, Yuyu Chen, Sijia Dai, Xiaowei He, Yunfeng Zou, Cheng Xu

**Affiliations:** 1 Department of Social Medicine, School of Public Health, Guangxi Medical University, Nanning, Guangxi, China; 2 Department of Toxicology, School of Public Health, Guangxi Medical University, Nanning, Guangxi, China; 3 Editorial Office of the Journal of Guangxi Medical University, Guangxi Medical University, Nanning, Guangxi, China

**Keywords:** employment guidance, employment satisfaction, graduates, importance-performance analysis (IPA), public health and preventive medicine

## Abstract

**Background:**

In recent years, the training for public health and preventive medicine graduates has expanded, and job positions have been reorganized, which may affect graduates’ job matching and subjective experiences. However, systematic evidence remains limited. This study evaluates the level, structure, and determinants of employment satisfaction among Guangxi Medical University graduates from 2021 to 2024, and suggests priorities for improvement based on these findings.

**Methods:**

We surveyed graduates online from February to August 2025 using a cross-sectional design. Of 243 submitted questionnaires, 49 had the same response to all 17 dimension-specific satisfaction items and were excluded. We analyzed the remaining 194 questionnaires; a separate 5-point item measured overall employment and development satisfaction. We examined associations using proportional odds ordinal logistic regression and used the 17 dimension-specific items for an exploratory Importance-Performance Analysis (IPA). Supplementary checks included prespecified model diagnostics, bootstrap analyses, alternative outcome coding, exclusion of respondents in further study or postdoctoral positions, and false-discovery-rate adjustment of univariable tests.

**Results:**

Overall satisfaction was low for 5.7% of respondents, average for 39.7%, and relatively high or very high for 54.6%. In the primary model, older age was associated with higher satisfaction (adjusted OR = 1.17, 95% CI 1.02–1.34; *p* = 0.027). Respondents who rated university career guidance as neutral or not helpful had lower odds of higher satisfaction (adjusted OR = 0.46, 95% CI 0.26–0.81; *p* = 0.008). Both associations had the same direction in the three-level ordinal model and after excluding 25 respondents in further study or postdoctoral positions. The exploratory IPA identified career development opportunities, the contribution of internships or practica to employability, and effort-reward fairness as areas for further study. Several quadrant assignments, however, had limited bootstrap stability.

**Conclusion:**

Just over half of respondents reported relatively high or very high satisfaction. Older age and more favorable retrospective ratings of university career guidance were associated with higher satisfaction. The cross-sectional data cannot establish the direction of these relationships or support causal conclusions. The exploratory IPA classifications require confirmation in larger, multicenter samples before they are used to guide interventions.

## Introduction

In recent years, the number of postgraduate students in public health and preventive medicine in China has continued to rise, along with higher recruitment standards and shifts in the job market. Rapid supply growth and improved quality may change how graduates find jobs and their employment experiences, potentially affecting job satisfaction. However, systematic evidence on this group’s satisfaction remains limited. Existing international studies show that public health graduates often work in non-governmental organizations and universities, and their overall satisfaction remains relatively high (1). Another study found that personal, social, and economic resources are related to job satisfaction (2). Still, because of China’s unique context regarding unit establishment, employment systems, regional development levels, and living costs, applying these findings directly has limitations. Research on public health postgraduates in China remains limited.

In the Chinese context, job satisfaction is influenced not only by job characteristics but also by factors such as the nature of the employer, whether the position is part of the establishment, regional attractiveness, cost of living, promotion and assessment mechanisms, and support for training and research. These institutional factors collectively play a role (3–8). Additionally, there are differences in training goals and employment paths between academic and professional graduate students (9, 10), which can lead to notable differences in subjective feelings like “sense of security,” “development expectations,” and “work-life balance,” even when salaries are the same. These factors are likely key variables affecting satisfaction, making data-based evaluations in the Chinese context an urgent need.

Accordingly, this article examines public health and preventive medicine graduates from Guangxi Medical University in the 2021 to 2024 cohorts, assessing overall and dimensional employment satisfaction. It identifies independent influencing factors after controlling for key background variables. Methodologically, we use ordered Logit/multivariate regression to model overall satisfaction and each dimension, and introduce Importance-Performance Analysis (IPA): using the standardized coefficient of the “overall satisfaction” regression model to define the “importance” of each item, and using the mean of each item to measure “performance.” This approach divides items into four quadrants and determines a priority improvement list based on “low performance × high importance,” providing operational suggestions for institutions and employers. This study is a single-university cross-sectional analysis, so approach extrapolation with caution. Nonetheless, it offers quantitative references for talent development, recruitment, and retention strategies at similar institutions and employers, and provides a methodological pathway for developing future monitoring indicator systems.

## Methods

### Research design and participants

We conducted a cross-sectional survey from February to August 2025 in the Guangxi Zhuang Autonomous Region. The target population was master’s and doctoral graduates of the Public Health and Preventive Medicine program at Guangxi Medical University from the 2021–2024 cohorts. We recruited a convenience sample through grade and class WeChat groups. Eligible graduates had a master’s or doctoral degree in Public Health or Preventive Medicine, had graduated between 2021 and 2024, and agreed to participate voluntarily with electronic informed consent. Of 300 invited graduates, 243 submitted questionnaires (response rate, 81.0%). Before outcome modeling, we classified questionnaires as low quality if respondents selected the same option for all 17 dimension-specific satisfaction items. Forty-nine questionnaires met this criterion; we included the remaining 194 in the primary analysis (64.7% of those invited). No additional questionnaires were excluded as duplicate submissions in the analytic dataset. The Ethics Review Committee of Guangxi Medical University approved the study (KY20250399). All participants provided electronic informed consent. The complete questionnaire is provided in Supplementary Table S1.

### Outcome variable

The questionnaire included 17 dimension-specific items: compensation and benefits; career growth potential; work environment; professional or role fit; workload intensity; career development opportunities; effort-reward fairness; work-life balance; working hours and job stress; leadership style and team collaboration; team or department coordination and coworker relations; organizational support for research or practice projects; usefulness of graduate coursework; contribution of internships or practica to employability; promotion pathways; societal respect for the profession; and personal accomplishment. A separate eighteenth item measured overall employment and development satisfaction and served as the regression outcome. Response options for each item were very dissatisfied, relatively dissatisfied, average, relatively satisfied, and very satisfied, scored 1–5. We defined the three descriptive categories from the separate overall item alone, without summing or averaging the 18 items. Respondents in further study or postdoctoral positions rated their current post-graduation training or postdoctoral position.

### Questionnaire development and measurement properties

We developed the questionnaire in Chinese for this survey to assess employment and career-development domains relevant to Public Health and Preventive Medicine graduates. We did not translate it from another language or independently validate it. We did not calculate a formal content-validity index or conduct a separate pilot validation study. Therefore, the questionnaire cannot be described as a validated scale. In this sample, the 17 dimension-specific items had high internal consistency (Cronbach’s alpha = 0.936; corrected item-total correlations, 0.494–0.778). The Kaiser-Meyer-Olkin statistic was 0.916, and Bartlett’s test of sphericity was significant (chi-square = 2400.045, df = 136; *p* < 0.001). Exploratory parallel analysis retained three components, which does not support treating the items as a single unidimensional construct (Supplementary Tables S2A–C).

### Social demographic characteristics

Social demographic variables include age (a continuous variable), gender (female, male), ethnicity (Han, Zhuang, and others), father’s educational level (high school or technical school or below, college or above), mother’s educational level (high school or technical school or below, college or above), educational attainment (master’s degree, doctoral degree), medical qualification certificate (yes, no), publication of SCI papers during school years (yes, no), program type (academic, professional), current employment or post-graduation destination type (medical institution, CDC, education/research/further study, or other units), and perceived helpfulness of university career guidance (very helpful, somewhat helpful, neutral, or not helpful). The original questionnaire categories were recoded into four groups: (1) medical institutions, comprising hospitals and maternal and child health care institutions; (2) Centers for Disease Control and Prevention (CDC); (3) education/research/further study, comprising further study or postdoctoral positions and higher education institutions or research institutes; and (4) other units, comprising Party or government agencies, health supervision institutions, other public institutions, state-owned enterprises, private enterprises, foreign-funded or joint-venture enterprises, the armed forces, and other destinations. For analysis, the four original responses were dichotomized as very/somewhat helpful versus neutral/not helpful.

### Statistical analysis

For descriptive and univariable analyses, we grouped the separate overall satisfaction item as low (very or relatively dissatisfied; scores 1–2), average (score 3), or satisfied (relatively or very satisfied; scores 4–5). The primary multivariable model retained the original five-level outcome. We summarized continuous variables as medians and interquartile ranges and categorical variables as frequencies and percentages. Univariable comparisons used chi-square or Fisher’s exact tests for categorical variables and the Kruskal-Wallis test for age. The proportional odds ordinal logistic model included age, gender, ethnicity, parental education, degree, medical qualification, SCI-indexed publication during graduate study, program type, current employment or post-graduation destination, and perceived helpfulness of university career guidance. Covariates were retained for their conceptual relevance; univariable *p* values were not the sole basis for selection. We report adjusted odds ratios (ORs), 95% confidence intervals, and *p* values. We assessed the proportional odds assumption with the Brant test, recognizing that a nonsignificant result does not establish that the assumption holds. Model checks included the number of complete cases, convergence, log-likelihood, AIC, BIC, design-matrix rank, and the standardized condition number. We assessed coefficient stability with 500 nonparametric bootstrap resamples.

We used the 17 dimension-specific items in an exploratory IPA. Standardized coefficients from a mutually adjusted proportional odds model provided model-derived indicators of relative importance, and item means measured performance. The dimension-specific items and overall satisfaction came from the same instrument and assessed related constructs. Shared variance, overlapping item content, and common-method dependence may therefore affect the IPA. We examined dependence and sampling stability using Spearman correlations, variance inflation factors, and 300 bootstrap resamples. We interpreted the quadrant assignments as exploratory classifications that require further evaluation before informing intervention priorities.

We compared included and excluded respondents on the measured characteristics. To examine the questionnaire’s measurement properties, we calculated Cronbach’s alpha, corrected item-total correlations, KMO, and Bartlett’s test, and conducted exploratory parallel analysis. Sensitivity analyses used a three-level ordinal outcome (scores 1–2, 3, and 4–5) and repeated the five-level model after excluding respondents in further study or postdoctoral positions. We also calculated Benjamini-Hochberg false-discovery-rate q values for the 11 univariable screening tests. All tests were two-sided. We used R 4.4.3 for the original models and independently verified scripts for the additional analyses.

## Results

### Sample characteristics

Of the 300 invited graduates, 243 submitted questionnaires (81.0%), and 194 entered the primary analysis (64.7% of those invited). Overall satisfaction was low for 11 participants (5.7%), average for 77 (39.7%), and relatively high or very high for 106 (54.6%; Table 1). Median age was 28 years, with an observed range of 23–41 years. We found no statistically significant differences between included and excluded respondents in the measured sociodemographic, educational, destination, or career-guidance variables (all *p* > 0.05; Supplementary Table S3). Selection related to unmeasured characteristics remains possible.

**Table 1:** Participant characteristics and univariable associations with overall satisfaction.

Characteristic	Low satisfaction	Average satisfaction	High satisfaction	*p* value
Participants, *n*	11	77	106	
Age, years, median (IQR)	27 (2)	28 (2)	28 (3)	0.014
Gender, *n* (%)				0.367
Male	3 (5.3)	27 (47.4)	27 (47.4)	
Female	8 (5.8)	50 (36.5)	79 (57.7)	
Ethnicity, *n* (%)				0.346
Han	6 (4.5)	59 (44.4)	68 (51.1)	
Zhuang	4 (8.7)	14 (30.4)	28 (60.9)	
Others	1 (6.7)	4 (26.7)	10 (66.7)	
Father’s educational level, *n* (%)				0.444
High school or technical school or below	9 (5.7)	66 (41.8)	83 (52.5)	
College or above	2 (5.6)	11 (30.6)	23 (63.9)	
Mother’s educational level, *n* (%)				0.275
High school or technical school or below	10 (6.0)	70 (41.7)	88 (52.4)	
College or above	1 (3.8)	7 (26.9)	18 (69.2)	
Educational attainment, *n* (%)				0.991
Master’s degree	10 (5.7)	69 (39.7)	95 (54.6)	
Doctoral degree	1 (5.0)	8 (40.0)	11 (55.0)	
Medical qualification certificate, *n* (%)				0.044
Yes	1 (2.4)	11 (26.2)	30 (71.4)	
No	10 (6.6)	66 (43.4)	76 (50.0)	
SCI-indexed publication during graduate study, *n* (%)				0.114
Yes	4 (4.2)	45 (46.9)	47 (49.0)	
No	7 (7.1)	32 (32.7)	59 (60.2)	
Current employment or post-graduation destination type, *n* (%)				0.415
Medical institution	8 (8.9)	38 (42.2)	44 (48.9)	
Centers for disease control and prevention	0 (0.0)	13 (36.1)	23 (63.9)	
Education/research/further study	3 (5.7)	20 (37.7)	30 (56.6)	
Other units	0 (0.0)	6 (40.0)	9 (60.0)	
Perceived helpfulness of university career guidance, *n* (%)				0.006
Neutral/not helpful	6 (7.0)	44 (51.2)	36 (41.9)	
Very/somewhat helpful	5 (4.6)	33 (30.6)	70 (64.8)	
Program type, *n* (%)				0.983
Academic degree program	7 (5.8)	48 (40.0)	65 (54.2)	
Professional degree program	4 (5.4)	29 (39.2)	41 (55.4)	

*N* = 194. Low satisfaction combines scores 1–2, average satisfaction is score 3, and high satisfaction combines scores 4–5 of the separate overall employment and development satisfaction item. Age is shown as median (interquartile range [IQR]); categorical data are *n* (row %). Percentages are calculated within each characteristic category and may not sum to 100 because of rounding. *p* values are unadjusted univariable comparisons using the Kruskal-Wallis test for age and chi-square or Fisher exact tests for categorical variables, as described in Methods. All 11 tests had Benjamini-Hochberg false-discovery-rate q values > = 0.05 (Supplementary Table S5); these comparisons are exploratory. SCI, Science Citation Index.

### Overall satisfaction level

Of 194 questionnaires, the average score for overall feelings about employment and development was 3.60 (Supplementary Table S4). Among the 17 items, the top three with the highest satisfaction rates were cooperation within team/department coordination and coworker relations, with an average score of 3.85 and a satisfaction rate of 71.1%. The work environment received an average score of 3.79 with a satisfaction rate of 69.1%. Professional/role fit had an average score of 3.61 and a satisfaction rate of 59.3%. The three areas with the highest dissatisfaction rates were: the impact of internship or practice experience on employability, with an average score of 3.33 and a dissatisfaction rate of 15.0%; working hours and job stress, with an average score of 3.34 and a dissatisfaction rate of 20.1%; and workload/work intensity, with an average score of 3.43 and a dissatisfaction rate of 15.9%.

The 17 dimension-specific items had high internal consistency (Cronbach’s alpha = 0.936), with corrected item-total correlations of 0.494–0.778. KMO was 0.916, and Bartlett’s test was significant (chi-square = 2400.045, df = 136; *p* < 0.001). Parallel analysis retained three components. We therefore did not treat the items as a single unidimensional scale (Supplementary Tables S2A–C).

### Univariable comparisons

Age (*p* = 0.014), medical qualification (*p* = 0.044), and perceived helpfulness of university career guidance (*p* = 0.006) differed across the three descriptive satisfaction groups in univariable analyses (Table 1). None of the 11 tests met q < 0.05 after Benjamini-Hochberg correction (age q = 0.077; career-guidance helpfulness q = 0.066; Supplementary Table S5). We treated these comparisons as exploratory and did not select covariates solely on their basis.

### Multivariate model

The Brant test did not reject the proportional odds assumption (omnibus chi-square = 21.16, *p* = 0.997; Supplementary Table S6). Sparse lower outcome categories limit the test’s power, so the high *p* value does not establish that the assumption holds. In the adjusted proportional odds model, each additional year of age was associated with higher odds of being in a higher satisfaction category (OR = 1.17, 95% CI 1.02–1.34; *p* = 0.027). The estimate describes a one-year age difference within this young graduate sample, whose observed ages ranged from 23 to 41 years. Respondents who rated university career guidance as neutral or not helpful had lower odds of higher satisfaction than those who rated it very or somewhat helpful (adjusted OR = 0.46, 95% CI 0.26–0.81; *p* = 0.008; Table 2). No other covariate was statistically significant. All 194 records had complete data for the model variables. The model converged with a full-rank design matrix and a standardized condition number of 3.07. Bootstrap estimates retained the observed direction in 99.4% of resamples for age and 99.2% for career guidance (Supplementary Table S7). These checks assess numerical stability within the sample. They cannot resolve concerns about measurement validity, selection bias, residual confounding, causality, or generalizability.

**Table 2:** Primary five-level proportional odds model of overall satisfaction.

Characteristic/comparison	Adjusted OR	95% CI lower	95% CI upper	*p* value
Age, per year	1.17	1.02	1.34	0.027
Gender				
Female vs. Male	1.28	0.69	2.38	0.432
Ethnicity				
Zhuang vs. Han	1.27	0.65	2.48	0.486
Others vs. Han	2.04	0.70	6.01	0.194
Father’s educational level				
College or above vs. high school or technical school or below	1.10	0.39	3.09	0.858
Mother’s educational level				
College or above vs. high school or technical school or below	1.93	0.61	6.17	0.265
Educational attainment				
Doctoral degree vs. master’s degree	0.99	0.35	2.83	0.987
Medical qualification certificate				
Yes vs. no	1.30	0.65	2.59	0.454
SCI-indexed publication during graduate study				
Yes vs. no	0.84	0.46	1.56	0.588
Program type				
Professional vs. academic	0.96	0.52	1.77	0.901
Current employment or post-graduation destination type				
CDC vs. medical institution	1.85	0.87	3.95	0.110
Education/research/further study vs. medical institution	0.99	0.47	2.06	0.974
Other units vs. medical institution	1.59	0.57	4.46	0.380
Perceived helpfulness of university career guidance				
Neutral/not helpful vs. very/somewhat helpful	0.46	0.26	0.81	0.008

*N* = 194 complete cases. The dependent variable is the original five-level overall employment and development satisfaction score (1–5), not the three descriptive groups in Table 1. All listed covariates were entered simultaneously. OR > 1 indicates higher odds of being in a higher satisfaction category. Age is modeled per additional year. OR, odds ratio; CI, confidence interval; CDC, Centers for Disease Control and Prevention; SCI, Science Citation Index. Reference categories were male; Han ethnicity; high school/technical secondary school or below for parental education; master’s degree; no medical qualification certificate; no SCI-indexed publication during graduate study; academic program; medical institution; and very/somewhat helpful university career guidance. The education/research/further study category includes respondents in further study or postdoctoral positions. The three-level outcome sensitivity model is reported in Supplementary Table S12; the analysis excluding the 25 respondents in further study or postdoctoral positions is reported in Supplementary Table S13. The estimates above remain those of the primary five-level model.

### IPA analysis

The IPA placed personal accomplishment, societal respect, compensation and benefits, and team or department coordination and coworker relations in the maintain-strengths quadrant. Career development opportunities, contribution of internships or practica to employability, and effort-reward fairness fell in the candidate-priorities quadrant (Figure 1; Supplementary Table S8). The 17 item scores showed moderate dependence: the largest absolute Spearman correlation was 0.773, and VIFs ranged from 2.25 to 4.15. All 300 bootstrap models converged. The original quadrant was retained in 68.0% of resamples for career development opportunities, 56.7% for internships or practica, and 42.3% for effort-reward fairness. Several maintain-strengths items also had limited stability. These classifications remain hypotheses for further evaluation and do not establish intervention priorities (Supplementary Table S9).

**Figure 1:**
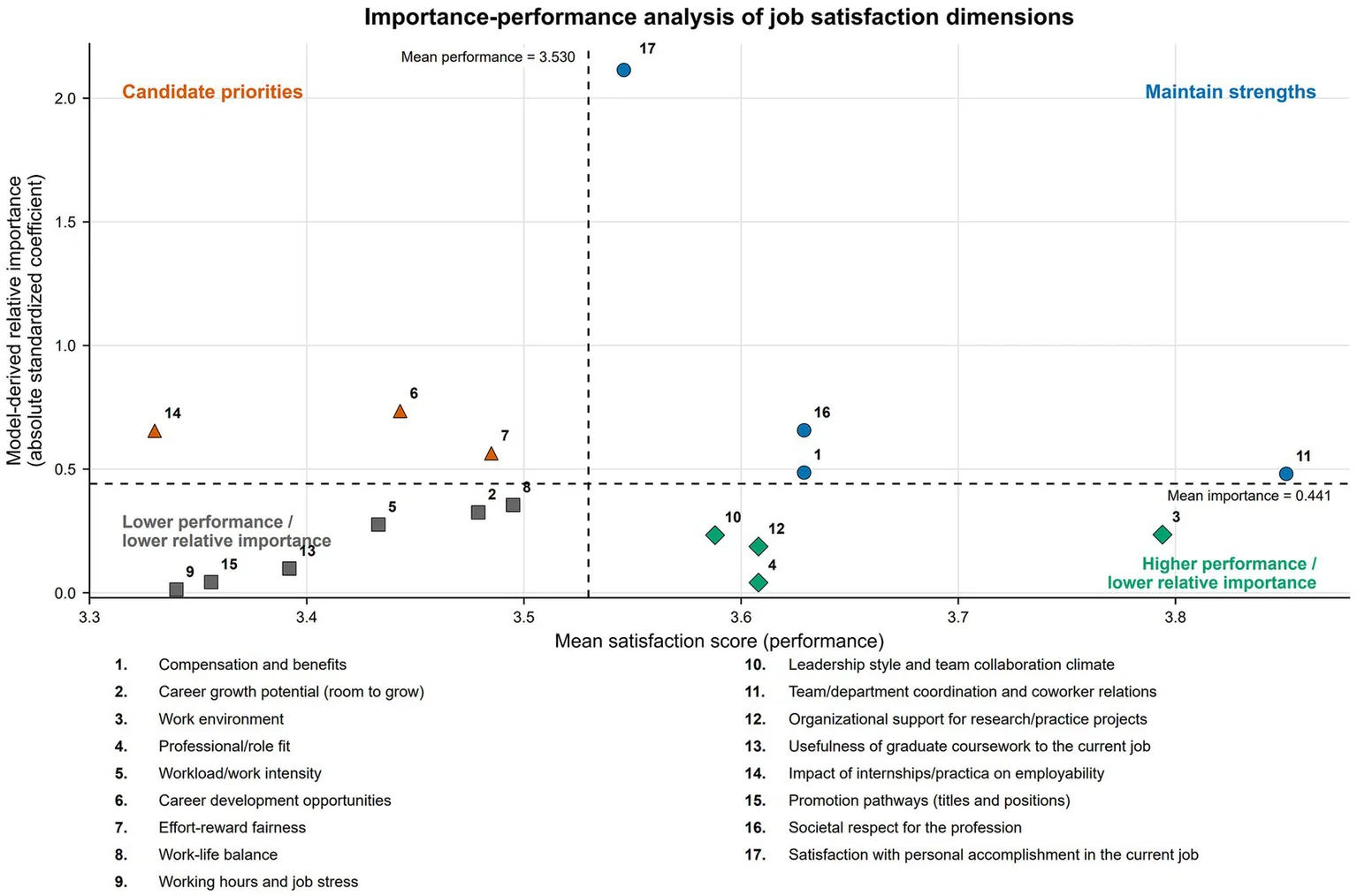
Importance-performance analysis (IPA) of job satisfaction dimensions. Each point represents one facet of job satisfaction, with the x-axis showing mean satisfaction (performance) and the y-axis showing absolute standardized coefficients derived from a multivariable proportional odds model, used as exploratory relative-importance indicators from the ordered logit model. The vertical and horizontal lines indicate the overall mean values that divide the matrix into four quadrants: “Maintain Strengths,” “Candidate priorities,” “Higher performance/lower relative importance,” and “Lower Priority.” Four items fall into the Maintain Strengths quadrant-personal accomplishment (3.55, 2.11), societal respect (3.63, 0.657), compensation and benefits (3.63, 0.486), and team/department coordination and coworker relations (3.85, 0.481)-indicating both high satisfaction and high model-derived relative importance. Three items are classified as Candidate priorities-career development opportunities (3.44, 0.735), impact of internships/practica on employability (3.33, 0.655), and effort–reward fairness (3.48, 0.564)-showing lower-than-average satisfaction but higher importance. The remaining dimensions are distributed across the Lower Priority and Higher performance/lower relative importance quadrants; the quadrant labels follow the conventional IPA framework and should not be interpreted as direct evidence of causal effects or actual overinvestment of resources.

### Sensitivity analysis

The expanded-sample and binary-outcome analyses gave age and career-guidance estimates in the same directions as the primary model (Supplementary Tables S10, S11). In the additional three-level ordinal model (scores 1–2, *n* = 11; score 3, *n* = 77; scores 4–5, *n* = 106), older age was associated with higher satisfaction (adjusted OR = 1.253, 95% CI 1.045–1.502; *p* = 0.015). Rating career guidance as neutral or not helpful was associated with lower satisfaction (adjusted OR = 0.428, 95% CI 0.233–0.787; *p* = 0.006; Supplementary Table S12). After excluding 25 respondents in further study or postdoctoral positions, the corresponding estimates were 1.261 (95% CI 1.073–1.483; *p* = 0.005) and 0.543 (95% CI 0.296–0.994; *p* = 0.048; Supplementary Table S13). Both additional models converged, had full-rank design matrices, and had standardized condition numbers below 3.2. The directions of the associations were consistent across analyses, although the confidence interval for career guidance in the restricted sample was close to the null.

## Discussion

In this sample from one university, 54.6% of respondents reported relatively high or very high overall satisfaction. Older age and more favorable retrospective ratings of university career guidance were associated with higher satisfaction after adjustment. Both associations had similar directions when we changed the outcome coding and excluded respondents in further study or postdoctoral positions. The data cannot establish causality. Graduates who were more satisfied with their current situation may have rated earlier career guidance more favorably; helpful guidance may also have contributed to their later experiences. Our cross-sectional design cannot distinguish between these explanations.

Previous studies offer context for these associations. Younger nurses may be particularly affected by mismatches between work expectations and available resources (11), and pharmacy graduates’ career readiness has been associated with job satisfaction (12). Studies of medical trainees and health professionals have linked satisfaction to recognition, time, income, workload, development pathways, and effort-reward balance (13–17). These findings give a basis for examining the domains in our survey. They do not establish that career guidance or an IPA-classified domain caused the satisfaction differences in this sample.

The IPA identified career development opportunities, the perceived contribution of internships or practica to employability, and effort-reward fairness as areas for further study. Relative importance was based on absolute standardized coefficients from a mutually adjusted model of correlated items, all collected together with overall satisfaction. Overlapping content, item redundancy, and common-method dependence may affect these estimates. Several quadrant assignments had moderate or low bootstrap stability, particularly effort-reward fairness. The classifications are therefore sample-dependent hypotheses; they do not show that reallocating resources to these areas would improve satisfaction. Studies of development opportunities, effort-reward balance, and physician satisfaction (18–21) provide context for further investigation but do not validate these classifications.

This study reports overall and dimension-specific satisfaction in an understudied graduate population, but its findings have several limitations. Convenience sampling at one medical university in Guangxi introduces selection bias and restricts generalizability. Training arrangements, public-sector employment structures, regional labor markets, and living costs may differ across provinces and universities. The comparison of included and excluded respondents was limited to measured variables and cannot rule out selection related to unmeasured characteristics. The cross-sectional design also prevents us from establishing temporal order or causality. Retrospective ratings of career guidance are particularly vulnerable to reverse causation and common-method bias. All measures were self-reported, raising concerns about recall, social-desirability, and response-style bias. The study-specific questionnaire had not been independently validated. Its high internal consistency does not establish construct validity, and parallel analysis suggested a multidimensional structure. Model estimation also had limitations. The two lowest overall-satisfaction categories contained only 5 and 6 participants, reducing precision and limiting the power of the Brant test. The three-level sensitivity analysis gave associations in the same directions, but its low-satisfaction group still contained only 11 participants. We estimated 14 predictor parameters from 194 participants. Model convergence, a full-rank matrix, low condition numbers, and bootstrap directional stability cannot rule out finite-sample bias, overfitting, or imprecision. Resampling the same participants does not establish external validity. The career-guidance estimate was also close to the null after excluding respondents in further study or postdoctoral positions. Confirmation will require larger, multicenter studies with probability-based sampling, longitudinal data, and independently validated measures.

Overall satisfaction was relatively high or very high in 54.6% of surveyed graduates. Older age and more favorable retrospective ratings of university career guidance were associated with higher satisfaction, although the direction of causality could not be determined. The exploratory IPA identified career development opportunities, the contribution of internships or practica to employability, and effort-reward fairness as areas for further study. These sample-dependent classifications require confirmation before they can guide resource allocation or intervention design.

## Data Availability

The original contributions presented in the study are included in the article/Supplementary material, further inquiries can be directed to the corresponding authors.
