# Supplementary material for: Employment satisfaction among public health and preventive medicine graduates and associated factors: a cross-sectional survey at Guangxi Medical University
Source: Front Public Health. 2026 Oct 1;14:1985186. doi: 10.3389/fpubh.2026.1985186 (PMC13668846; doi:10.3389/fpubh.2026.1985186)
Supplement: Supplementary file 1 [file Data_sheet_1.docx]

**Supplementary Data Sheet**

**Table S1. Study questionnaire, response options, instructions, and scoring**

| **Characteristics** | **Items** |
| --- | --- |
| Gender | * Male  * Female |
| Age | Years |
| Ethnicity | * Han  * Zhuang  * Other |
| Father’s highest educational level | * Primary school or below  * Junior high school  * Senior high school/technical secondary school  * Junior college  * Bachelor’s degree  * Master’s degree or above |
| Mother’s highest educational level | * Primary school or below  * Junior high school  * Senior high school/technical secondary school  * Junior college  * Bachelor’s degree  * Master’s degree or above |
| Degree level at graduation | * Master’s degree  * Doctoral degree |
| Your graduation date | Year |
| Before taking up your current employment, did you obtain any of the following professional qualification certificates? (Please select 1–8 items.) | * Physician qualification certificate  * Teacher qualification certificate  * Statistician qualification  * Nutritionist  * Psychological counselor  * Nurse qualification certificate  * Other  * None |
| During your time at university, what types of papers did you publish? (Please select 1–2 items.) | * None  * Chinese-language papers  * Papers indexed in SCI |
| When you applied for a graduate program in public health, was public health your first-choice major? | * Yes (a public health–related major was my first choice)  * No (I was transferred/adjusted into this major) |
| Major to which you were admitted in your master’s program | * Epidemiology and Health Statistics  * Health Toxicology  * Occupational Health and Environmental Health  * Nutrition and Food Hygiene  * Child and Adolescent Health and Maternal and Child Health  * Health Education and Health Promotion  * Medical Psychology  * Social Medicine and Health Service Management  * Professional Master of Public Health (MPH)  * Other |
| Current employment or post-graduation destination | * Further study / postdoctoral position  * Party or government agency  * Higher education institution/research institute  * Hospital  * Centers for Disease Control and Prevention (CDC)  * Maternal and child health care institution  * Health supervision institution  * Other public institution  * State-owned enterprise  * Private enterprise  * Foreign-funded / joint-venture enterprise  * Armed forces/military  * Other |
| In your opinion, how helpful was your university in providing career guidance for your job search? | * Very helpful  * Somewhat helpful  * Neutral  * Not helpful |
| Your satisfaction with your current job or current post-graduation position | For each of the following aspects, please rate your satisfaction with your current job or current post-graduation position.  Response options and scoring for each item: * Very dissatisfied = 1 * Relatively dissatisfied = 2 * Neutral = 3 * Relatively satisfied = 4 * Very satisfied = 5 |
| Items | Compensation and benefits |
|  | Career growth potential (room to grow) |
|  | Work environment |
|  | Professional/role fit |
|  | Workload/work intensity |
|  | Career development opportunities (specific opportunities) |
|  | Effort–reward fairness (is the return commensurate with effort?) |
|  | Work–life balance |
|  | Working hours and job stress |
|  | Leadership style and team collaboration climate |
|  | Team/department coordination and coworker relations |
|  | Organizational support for research/practice projects |
|  | Usefulness of graduate coursework to the current job |
|  | Impact of internships/practica on employability |
|  | Promotion pathways (titles and positions) |
|  | Societal respect for the profession |
|  | Satisfaction with personal accomplishment in the current job |
|  | Overall feeling about employment and development |

**Table S2A. Exploratory measurement summary for the 17 dimension-specific items**

| **Metric** | **Value** | **Interpretation/definition** |
| --- | --- | --- |
| Participants | 194.000 |  |
| Items | 17.000 | 17 dimension-specific items; overall satisfaction excluded |
| Cronbach alpha | 0.936 | Internal consistency; not proof of validity or unidimensionality |
| Omega total (one-component approximation) | 0.945 | Exploratory approximation based on the first principal component of Pearson correlations |
| KMO | 0.916 | Sampling adequacy |
| Bartlett chi-square | 2400.045 | df=136; p<0.001 |
| Parallel-analysis retained components | 3.000 | Observed eigenvalues exceeding 95th percentile of 1,000 random datasets |

**Table S2B. Corrected item-total correlations and alpha if item deleted**

| **Item** | **Mean** | **SD** | **Corrected item-total correlation** | **Cronbach's alpha if item deleted** |
| --- | --- | --- | --- | --- |
| Compensation and benefits | 3.629 | 0.856 | 0.651 | 0.933 |
| Career growth potential | 3.479 | 0.859 | 0.692 | 0.932 |
| Work environment | 3.794 | 0.881 | 0.739 | 0.931 |
| Professional or role fit | 3.608 | 0.934 | 0.630 | 0.933 |
| Workload intensity | 3.433 | 1.012 | 0.625 | 0.934 |
| Career development opportunities | 3.443 | 0.938 | 0.762 | 0.930 |
| Effort-reward fairness | 3.485 | 0.945 | 0.769 | 0.930 |
| Work-life balance | 3.495 | 0.978 | 0.645 | 0.933 |
| Working hours and job stress | 3.340 | 1.022 | 0.615 | 0.934 |
| Leadership style and team collaboration climate | 3.588 | 0.963 | 0.671 | 0.932 |
| Team or department coordination and coworker relations | 3.851 | 0.823 | 0.657 | 0.933 |
| Organizational support for research or practice projects | 3.608 | 0.934 | 0.671 | 0.932 |
| Usefulness of graduate coursework to the current position | 3.392 | 0.945 | 0.507 | 0.936 |
| Contribution of internships or practica to employability | 3.330 | 0.973 | 0.494 | 0.937 |
| Promotion pathways | 3.356 | 0.889 | 0.655 | 0.933 |
| Societal respect for the profession | 3.629 | 0.879 | 0.684 | 0.932 |
| Personal accomplishment in the current position | 3.546 | 0.876 | 0.778 | 0.930 |

**Table S2C. Parallel analysis of the 17 dimension-specific items**

| **Component** | **Observed eigenvalue** | **Random 95th percentile** | **Retain** |
| --- | --- | --- | --- |
| 1 | 8.592 | 1.655 | True |
| 2 | 1.840 | 1.519 | True |
| 3 | 1.465 | 1.412 | True |
| 4 | 0.921 | 1.328 | False |
| 5 | 0.576 | 1.256 | False |
| 6 | 0.530 | 1.188 | False |
| 7 | 0.483 | 1.130 | False |
| 8 | 0.391 | 1.072 | False |
| 9 | 0.354 | 1.019 | False |
| 10 | 0.333 | 0.969 | False |
| 11 | 0.308 | 0.915 | False |
| 12 | 0.262 | 0.866 | False |
| 13 | 0.246 | 0.817 | False |
| 14 | 0.213 | 0.770 | False |
| 15 | 0.178 | 0.722 | False |
| 16 | 0.156 | 0.668 | False |
| 17 | 0.152 | 0.609 | False |

**Table S3. Comparison of included and excluded questionnaires**

| **Variable** | **Category** | **Included (n=194)** | **Excluded (n=49)** | **Test** | **Statistic** | **P value** | **Effect size** | **Effect size type** |
| --- | --- | --- | --- | --- | --- | --- | --- | --- |
| Age, years | Median (IQR); range | 28 (2); 23-41 | 28 (2); 23-34 | Mann-Whitney U | 5338.500 | 0.175 | 0.123 | Rank-biserial correlation |
| Gender | Male | 57 (29.4%) | 14 (28.6%) | Chi-square | 0.000 | 1.000 | 0.000 | Cramer's V |
|  | Female | 137 (70.6%) | 35 (71.4%) |  |  |  |  |  |
| Ethnicity | Han | 133 (68.6%) | 32 (65.3%) | Chi-square | 1.548 | 0.461 | 0.080 | Cramer's V |
|  | Zhuang | 46 (23.7%) | 15 (30.6%) |  |  |  |  |  |
|  | Other | 15 (7.7%) | 2 (4.1%) |  |  |  |  |  |
| Father's educational level | High school/technical secondary school or below | 158 (81.4%) | 41 (83.7%) | Chi-square | 0.024 | 0.877 | 0.010 | Cramer's V |
|  | College or above | 36 (18.6%) | 8 (16.3%) |  |  |  |  |  |
| Mother's educational level | High school/technical secondary school or below | 168 (86.6%) | 43 (87.8%) | Chi-square | 0.000 | 1.000 | 0.000 | Cramer's V |
|  | College or above | 26 (13.4%) | 6 (12.2%) |  |  |  |  |  |
| Educational attainment | Master's degree | 174 (89.7%) | 41 (83.7%) | Chi-square | 0.862 | 0.353 | 0.060 | Cramer's V |
|  | Doctoral degree | 20 (10.3%) | 8 (16.3%) |  |  |  |  |  |
| Medical qualification certificate | No | 152 (78.4%) | 37 (75.5%) | Chi-square | 0.055 | 0.814 | 0.015 | Cramer's V |
|  | Yes | 42 (21.6%) | 12 (24.5%) |  |  |  |  |  |
| SCI-indexed paper during graduate study | No | 98 (50.5%) | 21 (42.9%) | Chi-square | 0.637 | 0.425 | 0.051 | Cramer's V |
|  | Yes | 96 (49.5%) | 28 (57.1%) |  |  |  |  |  |
| Program type | Academic program | 120 (61.9%) | 36 (73.5%) | Chi-square | 1.818 | 0.178 | 0.086 | Cramer's V |
|  | Professional program | 74 (38.1%) | 13 (26.5%) |  |  |  |  |  |
| Current employment or post-graduation destination | Medical institution | 90 (46.4%) | 16 (32.7%) | Chi-square | 4.075 | 0.253 | 0.129 | Cramer's V |
|  | CDC | 36 (18.6%) | 9 (18.4%) |  |  |  |  |  |
|  | Education/research/further study | 53 (27.3%) | 20 (40.8%) |  |  |  |  |  |
|  | Other units | 15 (7.7%) | 4 (8.2%) |  |  |  |  |  |
| Perceived helpfulness of university career guidance | Very/somewhat helpful | 108 (55.7%) | 31 (63.3%) | Chi-square | 0.638 | 0.425 | 0.051 | Cramer's V |
|  | Neutral/not helpful | 86 (44.3%) | 18 (36.7%) |  |  |  |  |  |

**Table S4. Item-level satisfaction distributions and mean scores**

| **Item / Response options** | **Very dissatisfied (1)** | **Relatively dissatisfied (2)** | **Neutral (3)** | **Relatively satisfied (4)** | **Very satisfied (5)** | **Mean score** |
| --- | --- | --- | --- | --- | --- | --- |
| Compensation and benefits | 4 (2.0%) | 10 (5.2%) | 66 (34.0%) | 88 (45.4%) | 26 (13.4%) | 3.63 |
| Career growth potential (room to grow) | 4 (2.1%) | 13 (6.7%) | 85 (43.8%) | 70 (36.1%) | 22 (11.3%) | 3.48 |
| Work environment | 3 (1.6%) | 12 (6.2%) | 45 (23.2%) | 96 (49.5%) | 38 (19.6%) | 3.79 |
| Professional/role fit | 8 (4.1%) | 9 (4.6%) | 62 (32.0%) | 87 (44.9%) | 28 (14.4%) | 3.61 |
| Workload/work intensity | 9 (4.6%) | 22 (11.3%) | 65 (33.5%) | 72 (37.1%) | 26 (13.4%) | 3.43 |
| Career development opportunities (specific opportunities) | 7 (3.6%) | 17 (8.8%) | 76 (39.2%) | 71 (36.6%) | 23 (11.9%) | 3.44 |
| Effort–reward fairness (is the return commensurate with effort?) | 5 (2.6%) | 24 (12.4%) | 60 (30.9%) | 82 (42.3%) | 23 (11.8%) | 3.48 |
| Work-life balance | 6 (3.1%) | 23 (11.9%) | 61 (31.4%) | 77 (39.7%) | 27 (13.9%) | 3.49 |
| Working hours and job stress | 8 (4.1%) | 31 (16.0%) | 66 (34.0%) | 65 (33.5%) | 24 (12.4%) | 3.34 |
| Leadership style and team collaboration climate | 4 (2.1%) | 22 (11.3%) | 56 (28.9%) | 80 (41.2%) | 32 (16.5%) | 3.59 |
| Team/department coordination and coworker relations | 2 (1.0%) | 8 (4.1%) | 46 (23.7%) | 99 (51.0%) | 39 (20.1%) | 3.85 |
| Organizational support for research/practice projects | 5 (2.6%) | 14 (7.2%) | 65 (33.5%) | 78 (40.2%) | 32 (16.5%) | 3.61 |
| Usefulness of graduate coursework to the current job | 9 (4.6%) | 15 (7.7%) | 82 (42.3%) | 67 (34.6%) | 21 (10.8%) | 3.39 |
| Impact of internships/practica on employability | 10 (5.2%) | 19 (9.8%) | 83 (42.8%) | 61 (31.4%) | 21 (10.8%) | 3.33 |
| Promotion pathways (titles and positions) | 5 (2.6%) | 20 (10.3%) | 89 (45.9%) | 61 (31.4%) | 19 (9.8%) | 3.36 |
| Societal respect for the profession | 5 (2.6%) | 10 (5.2%) | 64 (33.0%) | 88 (45.4%) | 27 (13.8%) | 3.63 |
| Satisfaction with personal accomplishment in the current job | 4 (2.1%) | 12 (6.2%) | 78 (40.2%) | 74 (38.1%) | 26 (13.4%) | 3.55 |
| Overall feeling about employment and development | 5 (2.6%) | 6 (3.1%) | 77 (39.7%) | 80 (41.2%) | 26 (13.4%) | 3.60 |

**Note.** Percentages are based on the 194 respondents included in the primary analysis.

**Table S5. Benjamini-Hochberg false-discovery-rate correction for univariable screening**

| **Variable** | **Original P value** | **BH-FDR q value** | **q<0.05** |
| --- | --- | --- | --- |
| Age | 0.014 | 0.077 | False |
| Gender | 0.367 | 0.543 | False |
| Ethnicity | 0.346 | 0.543 | False |
| Father's educational level | 0.444 | 0.543 | False |
| Mother's educational level | 0.275 | 0.543 | False |
| Educational attainment | 0.991 | 0.991 | False |
| Medical qualification certificate | 0.044 | 0.161 | False |
| SCI paper during graduate study | 0.114 | 0.314 | False |
| Current employment/post-graduation destination | 0.415 | 0.543 | False |
| Perceived helpfulness of university career guidance | 0.006 | 0.066 | False |
| Program type | 0.983 | 0.991 | False |

**Note.** The correction applies only to the 11 univariable screening tests; the adjusted multivariable model was prespecified to include all covariates.

**Table S6. Brant test for the proportional-odds assumption**

|  | **χ²** | **Degree of freedom** | **P value** |
| --- | --- | --- | --- |
| Omnibus | 21.16 | 42 | 0.997 |
| Age | 1.74 | 3 | 0.629 |
| Gender | 1.38 | 3 | 0.709 |
| Ethnicity (Zhuang vs Han) | 6.26 | 3 | 0.100 |
| Ethnicity (Others vs Han) | 0.75 | 3 | 0.861 |
| Father's educational level | 0.47 | 3 | 0.926 |
| Mother's educational level | 0.17 | 3 | 0.982 |
| Educational attainment | 2.14 | 3 | 0.543 |
| Medical qualification certificate | 1.59 | 3 | 0.661 |
| Publication of SCI papers during school | 5.77 | 3 | 0.123 |
| Program type | 1.06 | 3 | 0.786 |
| Type of employment unit (2 vs 1) | 0.22 | 3 | 0.975 |
| Type of employment unit (3 vs 1) | 1.87 | 3 | 0.600 |
| Type of employment unit (4 vs 1) | 0.32 | 3 | 0.957 |
| Perceived helpfulness of university career guidance (neutral/not helpful vs very/somewhat helpful) | 0.85 | 3 | 0.838 |

**Note.** A high P value does not prove the proportional-odds assumption, particularly with sparse lower outcome categories.

**Table S7. Bootstrap stability of the primary five-level proportional-odds model**

| **Predictor** | **Primary adjusted OR** | **Bootstrap median OR** | **Bootstrap 2.5th percentile** | **Bootstrap 97.5th percentile** | **Same-direction proportion (%)** | **Converged bootstrap models** | **Total bootstrap resamples** |
| --- | --- | --- | --- | --- | --- | --- | --- |
| Age (per year) | 1.17 | 1.182 | 1.037 | 1.37 | 99.4 | 500 | 500 |
| Female | 1.28 | 1.247 | 0.639 | 2.326 | 75.6 | 500 | 500 |
| Zhuang ethnicity | 1.27 | 1.252 | 0.583 | 2.716 | 69.8 | 500 | 500 |
| Other ethnicity | 2.04 | 2.018 | 0.491 | 6.822 | 82.2 | 500 | 500 |
| Father: college or above | 1.10 | 1.057 | 0.372 | 2.62 | 55.2 | 500 | 500 |
| Mother: college or above | 1.93 | 1.982 | 0.72 | 5.936 | 89.2 | 500 | 500 |
| Doctoral degree | 0.99 | 0.996 | 0.234 | 3.243 | 50.6 | 500 | 500 |
| Medical qualification certificate: yes | 1.30 | 1.364 | 0.645 | 2.627 | 81.6 | 500 | 500 |
| SCI paper during study: yes | 0.84 | 0.844 | 0.408 | 1.595 | 66 | 500 | 500 |
| Professional program | 0.96 | 0.96 | 0.496 | 1.845 | 54.4 | 500 | 500 |
| CDC | 1.85 | 1.919 | 0.867 | 4.821 | 94.6 | 500 | 500 |
| Education/research/further study | 0.99 | 0.992 | 0.412 | 2.211 | 50.4 | 500 | 500 |
| Other units | 1.59 | 1.602 | 0.589 | 5.01 | 83.2 | 500 | 500 |
| Career guidance rated neutral/not helpful | 0.46 | 0.453 | 0.242 | 0.805 | 99.2 | 500 | 500 |

**Note.** Reference categories match the primary model. All 500 bootstrap resamples converged.

**Table S8. Model-derived importance-performance analysis results**

| **Item** | **Mean** | **Importance** | **Quadrant** |
| --- | --- | --- | --- |
| Satisfaction with personal accomplishment in the current job | 3.55 | 2.11 | Maintain strengths |
| Career development opportunities (specific opportunities) | 3.44 | 0.735 | Candidate priorities |
| Societal respect for the profession | 3.63 | 0.657 | Maintain strengths |
| Impact of internships/practica on employability | 3.33 | 0.655 | Candidate priorities |
| Effort–reward fairness (is the return commensurate with effort?) | 3.48 | 0.564 | Candidate priorities |
| Compensation and benefits | 3.63 | 0.486 | Maintain strengths |
| Team/department coordination and coworker relations | 3.85 | 0.481 | Maintain strengths |
| Work–life balance | 3.49 | 0.354 | Low priority |
| Career growth potential (room to grow) | 3.48 | 0.325 | Low priority |
| Workload/work intensity | 3.43 | 0.276 | Low priority |
| Work environment | 3.79 | 0.235 | Higher performance/lower relative importance |
| Leadership style and team collaboration climate | 3.59 | 0.233 | Higher performance/lower relative importance |
| Organizational support for research/practice projects | 3.61 | 0.187 | Higher performance/lower relative importance |
| Usefulness of graduate coursework to the current job | 3.39 | 0.098 | Low priority |
| Promotion pathways (titles and positions) | 3.36 | 0.0431 | Low priority |
| Professional/role fit | 3.61 | 0.0416 | Higher performance/lower relative importance |
| Working hours and job stress | 3.34 | 0.0126 | Low priority |

**Note.** Quadrant classifications are exploratory and should not be interpreted as validated intervention priorities.

**Table S9. Diagnostic and bootstrap stability results for the importance-performance analysis**

| **Item** | **Mean performance** | **Signed standardized coefficient** | **Absolute standardized coefficient** | **95% CI lower** | **95% CI upper** | **P value** | **VIF** | **Original quadrant** | **Bootstrap same-direction proportion (%)** | **Bootstrap same-quadrant proportion (%)** |
| --- | --- | --- | --- | --- | --- | --- | --- | --- | --- | --- |
| Compensation and benefits | 3.629 | 0.486 | 0.486 | -0.121 | 1.092 | 0.117 | 2.331 | Maintain strengths | 92.7 | 39.3 |
| Career growth potential (room to grow) | 3.479 | 0.325 | 0.325 | -0.433 | 1.082 | 0.401 | 3.953 | Low priority | 77.3 | 58 |
| Work environment | 3.794 | -0.235 | 0.235 | -0.886 | 0.416 | 0.479 | 2.8 | Higher performance/lower relative importance | 70 | 70.3 |
| Professional/role fit | 3.608 | -0.041 | 0.041 | -0.647 | 0.564 | 0.893 | 2.248 | Higher performance/lower relative importance | 50 | 86 |
| Workload/work intensity | 3.433 | -0.276 | 0.276 | -0.992 | 0.44 | 0.45 | 3.244 | Low priority | 73 | 70.3 |
| Career development opportunities (specific opportunities) | 3.443 | 0.735 | 0.735 | -0.025 | 1.495 | 0.058 | 3.894 | Candidate priorities | 97 | 68 |
| Effort-reward fairness (is the return commensurate with effort?) | 3.485 | 0.564 | 0.564 | -0.142 | 1.269 | 0.117 | 3.325 | Candidate priorities | 92 | 42.3 |
| Work-life balance | 3.495 | 0.355 | 0.355 | -0.38 | 1.089 | 0.344 | 3.446 | Low priority | 77.3 | 45.7 |
| Working hours and job stress | 3.34 | -0.013 | 0.013 | -0.84 | 0.815 | 0.976 | 4.152 | Low priority | 47.7 | 72.3 |
| Leadership style and team collaboration climate | 3.588 | 0.233 | 0.233 | -0.414 | 0.879 | 0.481 | 2.709 | Higher performance/lower relative importance | 77 | 70.7 |
| Team/department coordination and coworker relations | 3.851 | 0.481 | 0.481 | -0.195 | 1.158 | 0.163 | 2.84 | Maintain strengths | 86 | 36.7 |
| Organizational support for research/practice projects | 3.608 | 0.187 | 0.187 | -0.413 | 0.787 | 0.542 | 2.503 | Higher performance/lower relative importance | 77.7 | 88 |
| Usefulness of graduate coursework to the current job | 3.392 | 0.098 | 0.098 | -0.547 | 0.744 | 0.766 | 2.44 | Low priority | 63.3 | 77 |
| Impact of internships/practica on employability | 3.33 | 0.655 | 0.655 | -0.018 | 1.327 | 0.056 | 2.646 | Candidate priority | 93.7 | 56.7 |
| Promotion pathways (titles and positions) | 3.356 | -0.043 | 0.043 | -0.685 | 0.598 | 0.895 | 2.28 | Low priority | 57.7 | 86.3 |
| Societal respect for the profession | 3.629 | 0.657 | 0.657 | 0.006 | 1.307 | 0.048 | 2.697 | Maintain strengths | 98.7 | 63.7 |
| Satisfaction with personal accomplishment in the current job | 3.546 | 2.114 | 2.114 | 1.366 | 2.863 | <0.001 | 3.456 | Maintain strengths | 100 | 67 |

**Note.** VIF denotes variance inflation factor. Bootstrap quadrant retention is based on 300 converged resamples.

**Table S10. Sensitivity analysis including all 243 respondents**

| **Variables** | **OR** | **95% lower limit** | **95% upper limit** | **P value** |
| --- | --- | --- | --- | --- |
| Age | 1.17 | 1.03 | 1.32 | 0.014 |
| Gender |  |  |  |  |
| Female vs Male | 1.58 | 0.91 | 2.75 | 0.106 |
| Ethnicity |  |  |  |  |
| Zhuang vs Han | 1.26 | 0.70 | 2.26 | 0.446 |
| Others vs Han | 2.39 | 0.84 | 6.80 | 0.103 |
| Father's educational level |  |  |  |  |
| College or above vs high school or technical school or below | 1.00 | 0.44 | 2.30 | 0.998 |
| Mother's educational level |  |  |  |  |
| College or above vs high school or technical school or below | 2.26 | 0.89 | 5.74 | 0.087 |
| Educational attainment |  |  |  |  |
| Doctoral degree vs master's degree | 0.66 | 0.26 | 1.63 | 0.366 |
| Medical qualification certificate |  |  |  |  |
| Yes vs no | 1.16 | 0.63 | 2.14 | 0.640 |
| Publication of SCI papers during school |  |  |  |  |
| Yes vs no | 1.06 | 0.61 | 1.85 | 0.830 |
| Program type |  |  |  |  |
| Professional vs academic | 1.06 | 0.61 | 1.85 | 0.840 |
| Current employment or post-graduation destination type |  |  |  |  |
| CDC vs medical institution | 2.19 | 1.11 | 4.31 | 0.024 |
| Education/research/further study vs medical institution | 1.56 | 0.80 | 3.03 | 0.189 |
| Other units vs medical institution | 1.85 | 0.74 | 4.64 | 0.189 |
| Assistance from the school's career guidance |  |  |  |  |
| Neutral/not helpful vs very/somewhat helpful | 0.39 | 0.23 | 0.65 | <0.001 |

**Note.** Odds ratios above 1 indicate higher odds of a higher satisfaction category.

**Table S11. Sensitivity analysis using a binary satisfaction outcome**

| **Variables** | **OR** | **95% lower limit** | **95% upper limit** | **P value** |
| --- | --- | --- | --- | --- |
| Age | 1.29 | 1.07 | 1.57 | 0.008 |
| Gender |  |  |  |  |
| Female vs Male | 1.43 | 0.70 | 2.93 | 0.329 |
| Ethnicity |  |  |  |  |
| Zhuang vs Han | 1.77 | 0.81 | 3.87 | 0.149 |
| Others vs Han | 1.82 | 0.52 | 6.30 | 0.347 |
| Father's educational level |  |  |  |  |
| College or above vs high school or technical school or below | 1.26 | 0.37 | 4.33 | 0.710 |
| Mother's educational level |  |  |  |  |
| College or above vs high school or technical school or below | 2.23 | 0.54 | 9.21 | 0.268 |
| Educational attainment |  |  |  |  |
| Doctoral degree vs master's degree | 1.01 | 0.31 | 3.33 | 0.988 |
| Medical qualification certificate |  |  |  |  |
| Yes vs no | 1.58 | 0.68 | 3.66 | 0.287 |
| Publication of SCI papers during school |  |  |  |  |
| Yes vs no | 0.60 | 0.29 | 1.23 | 0.165 |
| Program type |  |  |  |  |
| Professional vs academic | 0.96 | 0.46 | 1.97 | 0.902 |
| Current employment or post-graduation destination type |  |  |  |  |
| CDC vs medical institution | 1.76 | 0.72 | 4.27 | 0.213 |
| Education/research/further study vs medical institution | 1.23 | 0.53 | 2.86 | 0.637 |
| Other units vs medical institution | 1.61 | 0.48 | 5.36 | 0.440 |
| Assistance from the school's career guidance |  |  |  |  |
| Neutral/not helpful vs very/somewhat helpful | 0.40 | 0.21 | 0.75 | 0.005 |

**Note.** The binary outcome contrasts scores 4-5 with scores 1-3.

**Table S12. Sensitivity analysis using a three-level ordinal outcome**

| **Predictor** | **Adjusted OR** | **95% CI lower** | **95% CI upper** | **P value** |
| --- | --- | --- | --- | --- |
| Age (per year) | 1.253 | 1.045 | 1.502 | 0.015 |
| Female vs male | 1.320 | 0.676 | 2.574 | 0.416 |
| Zhuang vs Han | 1.531 | 0.719 | 3.262 | 0.269 |
| Other ethnicity vs Han | 1.655 | 0.481 | 5.697 | 0.424 |
| Father college+ vs lower | 1.157 | 0.345 | 3.882 | 0.813 |
| Mother college+ vs lower | 2.081 | 0.512 | 8.462 | 0.306 |
| Doctoral vs master's | 1.040 | 0.335 | 3.226 | 0.946 |
| Medical qualification yes vs no | 1.695 | 0.753 | 3.813 | 0.202 |
| SCI paper yes vs no | 0.752 | 0.384 | 1.470 | 0.404 |
| Professional vs academic | 0.999 | 0.507 | 1.971 | 0.998 |
| CDC vs medical institution | 1.921 | 0.825 | 4.475 | 0.130 |
| Education/research/further study vs medical institution | 1.238 | 0.551 | 2.781 | 0.605 |
| Other units vs medical institution | 1.820 | 0.581 | 5.698 | 0.304 |
| Neutral/not helpful guidance vs very/somewhat helpful | 0.428 | 0.233 | 0.787 | 0.006 |

**Note.** Outcome categories were scores 1-2, score 3, and scores 4-5.

**Table S13. Sensitivity analysis excluding respondents in further study or postdoctoral positions**

| **Predictor** | **Adjusted OR** | **95% CI lower** | **95% CI upper** | **P value** |
| --- | --- | --- | --- | --- |
| Age (per year) | 1.261 | 1.073 | 1.483 | 0.005 |
| Female vs male | 1.347 | 0.687 | 2.640 | 0.386 |
| Zhuang vs Han | 1.294 | 0.636 | 2.631 | 0.477 |
| Other ethnicity vs Han | 1.615 | 0.464 | 5.626 | 0.451 |
| Father college+ vs lower | 1.107 | 0.328 | 3.737 | 0.870 |
| Mother college+ vs lower | 1.738 | 0.465 | 6.501 | 0.411 |
| Doctoral vs master's | 0.328 | 0.052 | 2.052 | 0.233 |
| Medical qualification yes vs no | 1.204 | 0.584 | 2.484 | 0.615 |
| SCI paper yes vs no | 0.946 | 0.497 | 1.801 | 0.867 |
| Professional vs academic | 0.940 | 0.490 | 1.803 | 0.853 |
| CDC vs medical institution | 1.957 | 0.913 | 4.194 | 0.084 |
| Education/research/further study vs medical institution | 0.888 | 0.384 | 2.053 | 0.781 |
| Other units vs medical institution | 1.563 | 0.554 | 4.404 | 0.398 |
| Neutral/not helpful guidance vs very/somewhat helpful | 0.543 | 0.296 | 0.994 | 0.048 |

**Note.** This analysis excludes 25 respondents classified in further study or postdoctoral positions.
